# Ultrasound Based Planning and Navigation for Non-Anatomical Liver Resections – An *Ex-Vivo* Study

**DOI:** 10.1109/OJEMB.2019.2961094

**Published:** 2020-02-14

**Authors:** Iwan Paolucci, Raluca-Maria Sandu, Luca Sahli, Gian Andrea Prevost, Federico Storni, Daniel Candinas, Stefan Weber, Anja Lachenmayer

**Affiliations:** ARTORG Center for Biomedical Engineering ResearchUniversity of Bern 27210 Bern Switzerland; Department of Visceral Surgery and Medicine, Inselspital, Bern University HospitalUniversity of Bern 27210 Bern Switzerland

**Keywords:** Computer-assisted surgery, resection techniques, ultrasonography, liver neoplasms

## Abstract

*Goal:* Non-anatomical resections of liver tumors can be very challenging as the surgeon cannot use anatomical landmarks on the liver surface or in the ultrasound image for guidance. This makes it difficult to achieve negative resection margins (R0) and still preserve as much healthy liver tissue as possible. Even though image-guided surgery systems have been introduced to overcome this challenge, they are still rarely used due to their inaccuracy, time-effort and complexity in usage and setup. *Methods:* We have developed a novel approach, which allows us to create an intra-operative resection plan using navigated ultrasound. First, the surface is scanned using a navigated ultrasound, followed by tumor segmentation on a midsection ultrasound image. Based on this information, the navigation system calculates an optimal resection strategy and displays it along with the tracked surgical instruments. In this study, this approach was evaluated by three experienced hepatobiliary surgeons on ex-vivo porcine models. *Results:* Using this technique, an R0 resection could be achieved in 22 out of 23 (95.7% R0 resection rate) cases with a median resection margin of 5.9 mm (IQR 3.5–7.7 mm). The resection margin between operators 1, 2 and 3 was 7.8 mm, 4.15 mm and 5.1 mm respectively (p = 0.054). *Conclusions:* This approach could represent a useful tool for intra-operative guidance in non-anatomical resection alongside conventional ultrasound guidance. However, instructions and training are essential especially if the operator has not used an image-guidance system before.

## I. Introduction

Surgical resection is the current gold standard for curative care of primary and metastatic hepatic tumors. This procedure can be achieved by removing the segments containing the tumor, so called anatomical resections, or by non-anatomical, so-called atypical liver resections. The downside of anatomical resections is that also a large part of healthy liver tissue is removed. Recently, non-anatomical resections are becoming more popular, as they spare more healthy liver tissue than anatomical resections with similar oncological outcomes [1]–[3]. In a non-anatomical resection, only the tumor with a safety margin of 5–10 mm is resected leaving the patient with more healthy liver tissue allowing repeated resections in the future if necessary. Compared to anatomical resections, it is more challenging to keep a negative resection margin as anatomical landmarks cannot be used for intra-operative guidance [2]. This negative resection margin, commonly known as R0 resection, has been reported to be essential for good oncologic outcomes [4].

Non-anatomical resection is started by drawing the anticipated resection line onto the liver surface based on the intra-operative ultrasound. During the resection process, intra-operative ultrasound is used to confirm a safe distance to the tumor. Finally, once the depth is reached, the distance to the tumor is again confirmed on ultrasound and the tumor is removed. This is a challenging process which depends on the operator's ability and experience with mentally reconstructing the spatial relationships of the ultrasound image and the intra-operative scene.

To overcome these challenges, image-guidance systems have been introduced into the surgical workflow [5], [6]. These systems measure the pose of the surgical instruments and display their position on a virtual model of the anatomy. They mainly rely on a registration process to align a preoperative model with the patient's anatomy intraoperatively. This process is time-consuming, complex and error prone which is the main reason why such systems are rarely used [7]. Recent work focused on either simplifying the registration process [8] or correcting the deformations by incorporating biomechanical deformation models [9]–[12]. However, in clinical studies none of these approaches have proven to be accurate enough or applicable in a real life setting [6], [13].

Tissue deformation is the major obstacle, that needs to be overcome to successfully introduce an image-guided surgery system [13], [14]. Large deformations occur between the pre-operative image acquisition for the pre-operative model and the intra-operative field, especially after mobilization of the liver. Further deformations happen during the resection process and after each resection in cases with multiple tumors. This problem could be addressed using an image-guidance system relying only on intra-operative imaging. Such a system would acquire its image data after the large deformation happened – just before every resection. Initial studies have shown such approaches by providing means of 3D US compounding combined with visualization similar to multi planar viewers commonly used for CT imaging [15]–[17]. However, these systems do not incorporate resection planning functionality which is essential for non-anatomical resection to provide visual guidance through the liver tissue.

In this study, we have developed and evaluated a navigation approach, where navigated intra-operative US data is used to create a virtual model and a surgical plan on the spot. This technique does not require a separate registration process and it is only affected by deformations caused by the surgeon during the resection process. With this approach a virtual draft of the surgical plan is created, which serves as a guiding map through the procedure. We hypothesize that using such an intra-operative surgical sketch allows the surgeon to acquire negative resection margins.

A preliminary version of this work has been reported at the Annual Meeting of the German Society of Robotic and Computer Assisted Surgery (CURAC) 2019 [18].

## II. Materials and Methods

### A. System Overview

**Fig. 1.**
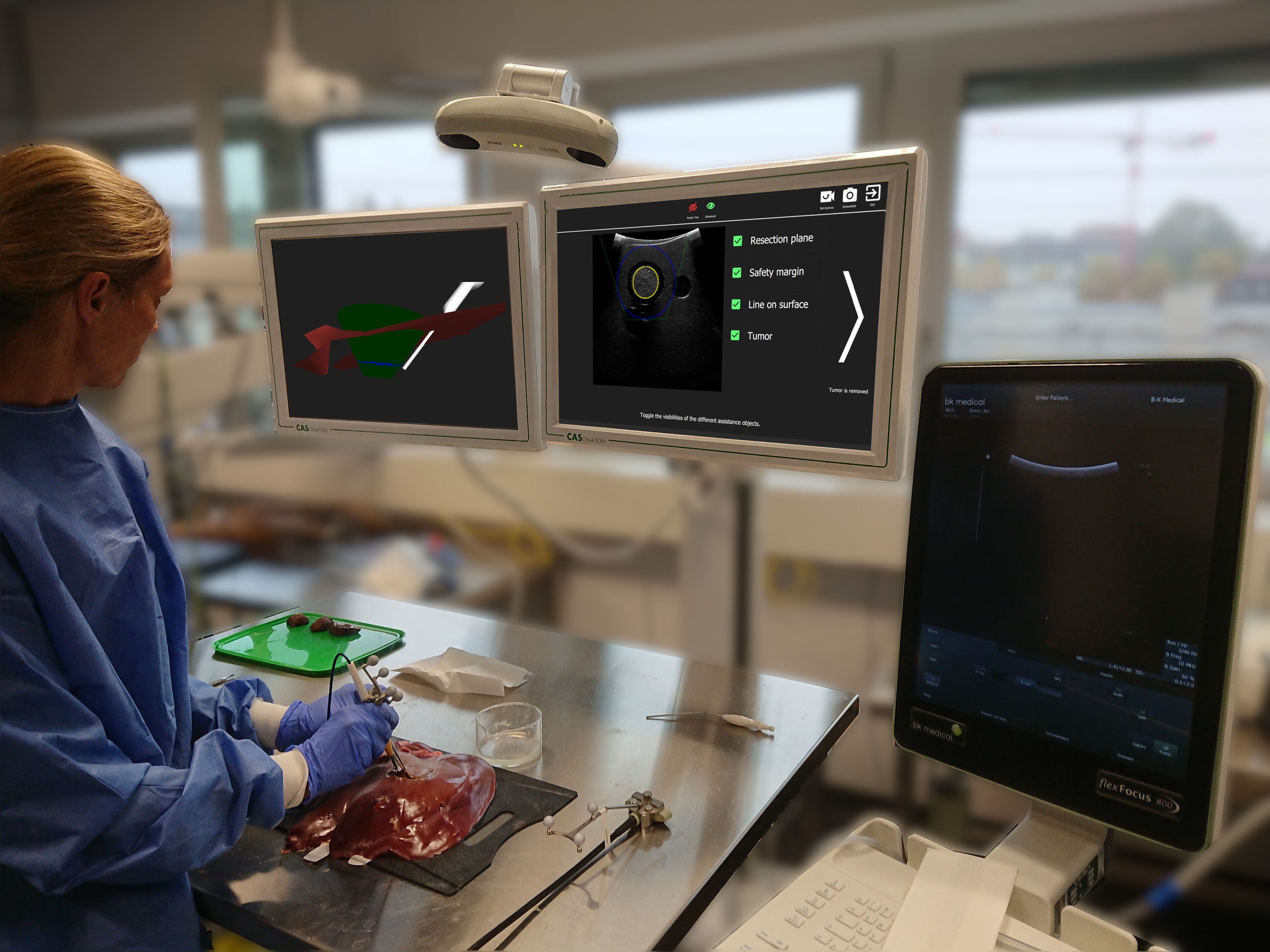
The navigation system in a demo setup with the optical tracking camera, the navigation screens, the navigated instruments and the operator.

The proposed system is based on the CAS-One (CAScination AG, Switzerland) navigation system for liver surgery, which is equipped with the Polaris (Northern Digital, Canada) optical tracking system and the FlexFocus 800 (BK Medical, Denmark) ultrasound (US) system (Fig. 1). The US probe is tracked with an optical marker shield and calibrated preoperatively using a z-wire phantom [19]. Another marker shield can be attached to any type of cylindrical instrument, such as pointer, electrocautery or CUSA, and calibrated with a dedicated calibration device [20].

For these experiments, a customized software, specifically designed for non-anatomical resections was developed and integrated into the navigation system. The software-workflow for creating such a surgical plan consists of the following steps, which are described in detail in the following sections

### B. Surface Scanning

A surface model of the liver is acquired by scanning the liver using the intra-operative US probe. A support-vector-machine(SVM)-based image classifier detects whether the US

**Fig. 2.**
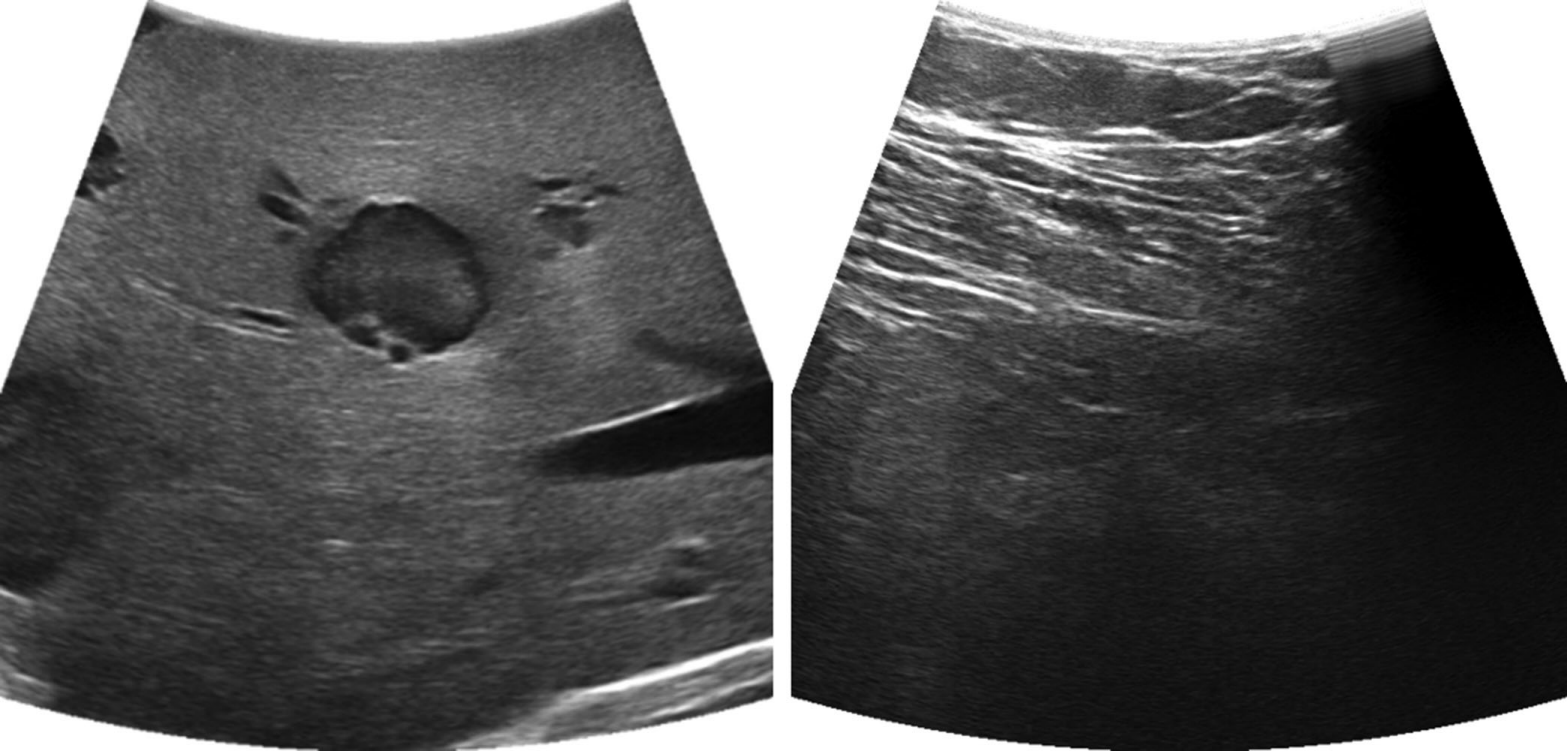
US images with contact to the liver (left), and contact to other tissue (right), Only the image with full contact to the liver will be classified as “contact image.”

Algorithm 1 **Data**: list of images *I*, list of transformations *T*, number of images *n*
**Result**: 3D mesh
**while** *i* ≤ *n* **do**
**if** I_i_ **not** on surface
clear buffer
**continue**
${p_i}{ \leftarrow ^C}{T_{MS}} \ast ^{{MS}}{T_{US}} \ast {T_{scale}}*{p_{origin}}$
add $p_{i}$ to *buffer*
**if** lof ($p_{i}$, *buffer*) > 1
add p_i_ to ptcloud
remove p_i-10_ from buffer
**end while**
calculate mesh from ptcloud
**return** mesh

probe is in contact with the liver surface [21] (Fig. 2). The minimum, maximum, mean, standard deviation and kurtosis of the upper third of the image are used as features. Time gain control was set to 0 and the automatic contrast adjustment function of the US device was used which automatically adjusts the B-mode gain, such that the image has a specific brightness. If the SVM detects that the image has contact to the liver, the current position of the US probe is recorded and added to a buffer. The local outlier factor (LOF), computed over the last 10 points, is used to detect whether a newly recorded point is an outlier [22]. If the LOF is below 1 the point is considered an inlier and added to the point cloud. From this point cloud, the virtual liver surface is reconstructed using the method described by Hoppe *et al.* [23]. A description in pseudocode is given in Algorithm 1 and a video of the surface scanning is provided in the supplementary material.

### C. Tumor Segmentation

**Fig. 3.**
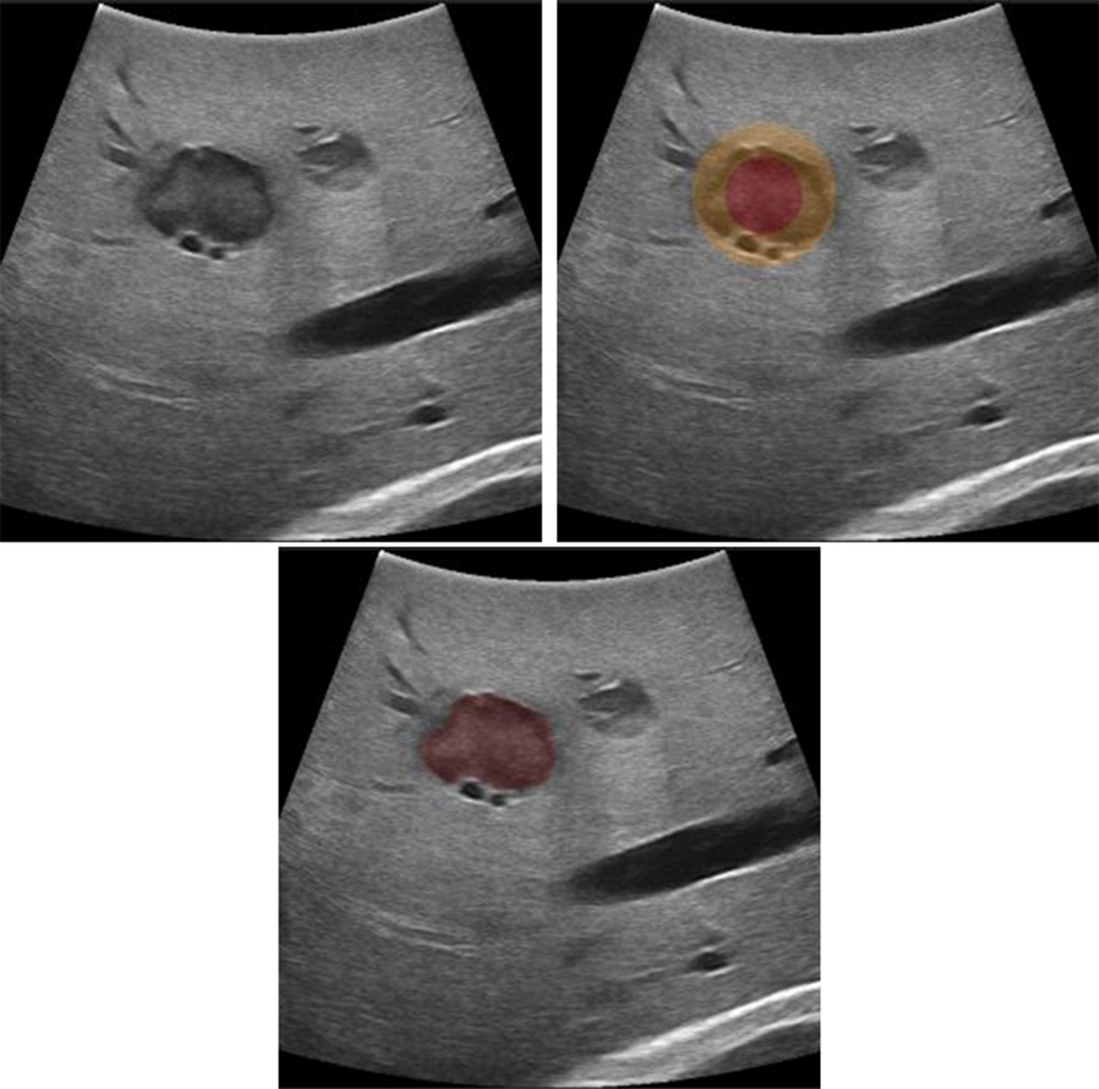
Segmentation of the tumor using the graph cuts algorithms. The algorithm is initialized with two overlapping circles red labelled as “definitely tumor”, orange as “probably foreground”, and everything outside as “background.”

In order to locate the tumor, a semi-automatic tumor segmentation method based on the “Graph Cuts” algorithm [24] is used. First, an US image with the largest diameter of the tumor is acquired and frozen. To initialize the tumor segmentation the operator clicks in the center of the tumor and selects an approximate size. A circle with the selected diameter is used as “foreground” for the segmentation initialization, and surrounding ring circle with a 20 mm margin is used as “probably foreground” (Fig. 3 top right). If needed, the segmentation result can be modified by clicking on the area which has to be added or removed from the segmentation mask. This can be done by simply clicking on the part which was over or under-segmented. The software automatically decides whether this area should be added or removed from the segmentation.

### D. Resection Planning

**Fig. 4.**
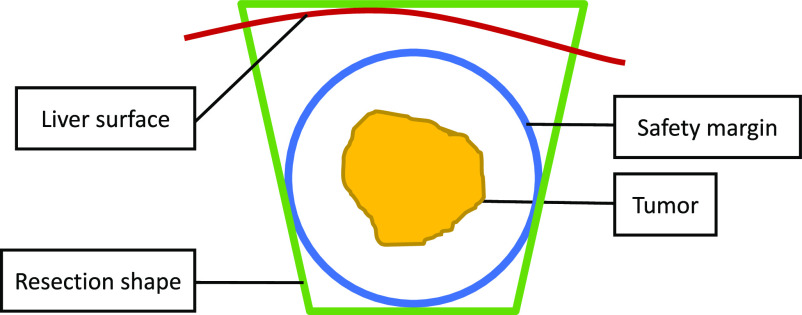
Concept of the planning method where a conical resection shape (green) is fitted around the safety margin (blue) of a tumor (yellow) with the closest distance to the surface (red).

For the resection planning, a user-chosen resection shape is fitted around the tumor with respect to the safety margin. Once the operator confirms the tumor segmentation, the center and diameter of the tumor are calculated. Ten the operator can then choose the desired resection margin and shape for this specific case. The software fits the resection shape into the model with respect to given shape and distance constraints (Fig. 4). The start of the resection line is calculated as the intersection of the resection shape with the liver surface.

### E. Visualization

**Fig. 5.**
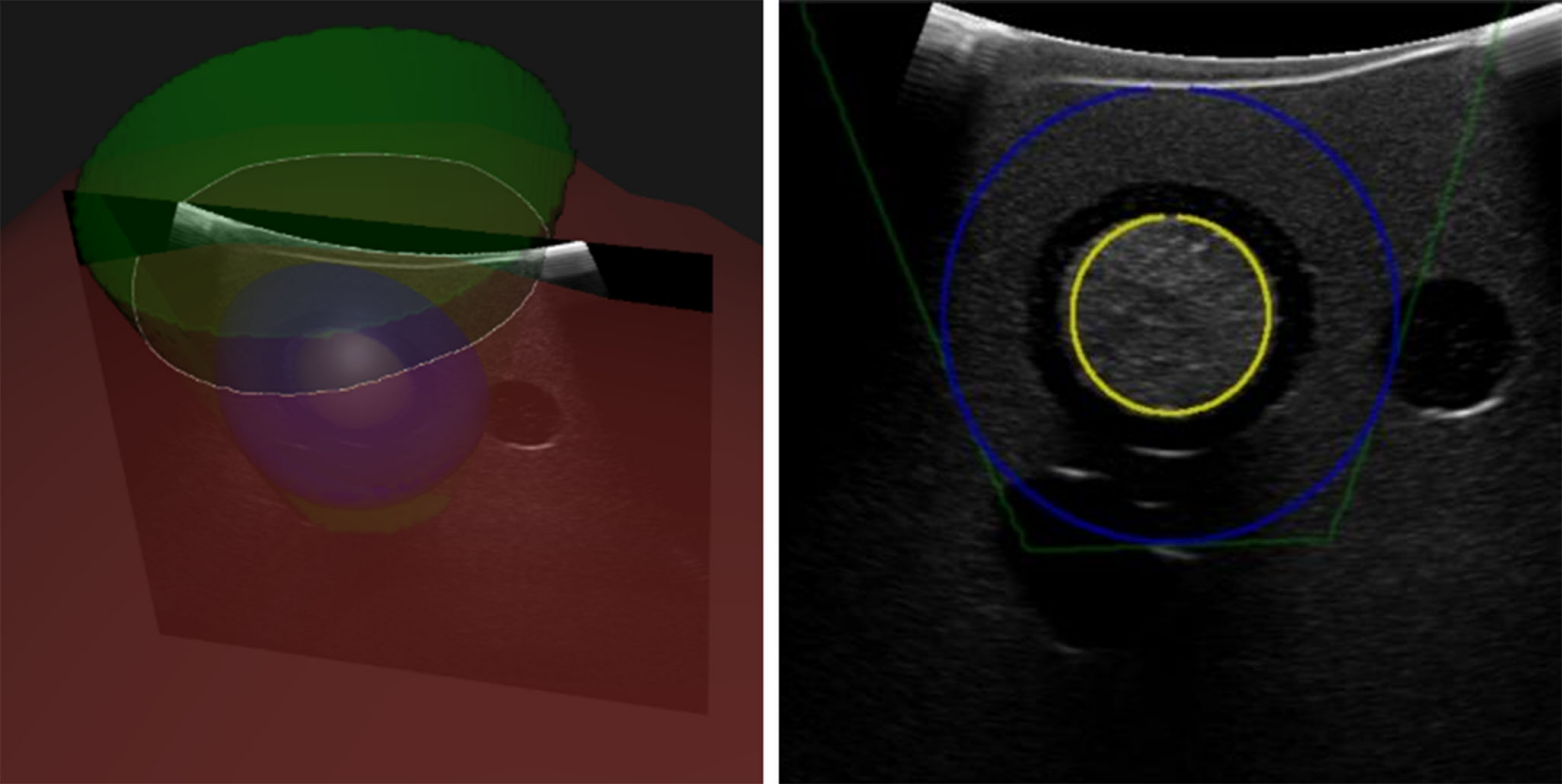
Visualization of the resection shape on the 3D screen (left) and as an overlay on the US image (right). All structures can be enabled or disabled separately.

The surgical plan is visualized on a 3D screen along with the tracked instruments. For each US frame, the 3D model is projected onto the US image plane and visualized as a semitransparent overlay (Fig. 5). The operator can separately enable or disable the visualization of the tumor, safety margin, cutting plane and cutting line.

### F. Experimental Evaluation

We evaluated the proposed navigation method on *ex-vivo* porcine livers with intrahepatic tumor mimics. The tumor mimics were made of 1% agar, 0.5% corn starch for US contrast and 0.5% barium sulfate for CT contrast. The tumors were randomly placed (2 per lobe) at depths of 1–2 cm in the liver by injection with a hypodermic needle. The lesions appeared as hyperechoic regions on the B-Mode US image. The mean size of the tumors was 12.1 mm (± 2.5 mm). For the experiment, 3 hepatobiliary surgeons were given an *ex-vivo* porcine liver containing 8 tumors each (total: n = 24), which they had to resect using the following workflow:

1) Preparing the instruments
2) Scanning the liver surface in the region of the tumor
3) Semi-automatic segmentation of the tumor
4) Selecting the desired resection shape and margin
5) Resecting the tumor using only the navigation screen

All resections were planned with a conical resection shape (Fig. 4) and a 10 mm safety margin, which is a common choice for resections of primary liver cancer. Based on this resection model, the navigation screen was used to resect the intrahepatic tumor (Fig. 5). To resect the tumors, we used an optically tracked monopolar electrocautery device (Medtronic, Ireland) with a scalpel electrode. During the resection procedure, the operator was not allowed to use the intra-operative ultrasound to avoid bias. First, a resection line was drawn on the liver surface using the navigation screen. Afterwards, the operator alternatively resected and double checked the path on the navigation screen. Finally, when the navigation screen indicated sufficient depth below the tumor, the operator cut the resection specimen out of the liver. A video of the process of resecting the tumor is provided in the supplementary material.

For margin analysis the resected specimens were labelled using the electrocautery device and immediately frozen to −15 °Celsius. Each frozen specimen was scanned with a CT scanner (Somatom, Toshiba, Japan) to visualize the resection margin. On this CT scan, the tumor and the resection specimen were segmented using Amira (Thermo Fisher Scientific, USA). The resection margin was measured in 3D as the distance between the tumor and the resection specimen. Every resection specimen was rated as an R0 or R1 resection where R0 resection was defined as a margin of >1 mm. Additionally, the time required to plan the resection (steps 2–4) and the time required for performing the resection were measured (step 5).

To the best of our knowledge, there is no similar study reporting resection rate in *ex-vivo* trials of liver navigation method. Therefore, we compared the R0 resection rate for this approach with the rates reported from clinical trials [2].

### G. Statistical Analysis

Descriptive statistics were used to report the R0-resection rates, resection margins and procedural times. Kruskal-Wallis test was used to test the inter-user variability on the resection margin and χ^2^-test was used to test the inter-user variability on the R0 resection rate. Dunn's test was used as post-hoc test following Kruskal-Wallis test [25]. All analyses were performed using the software R (R Foundation for Statistical Computing, Austria), and significance levels were set at p = 0.05.

## III. Results

**Fig. 6.**
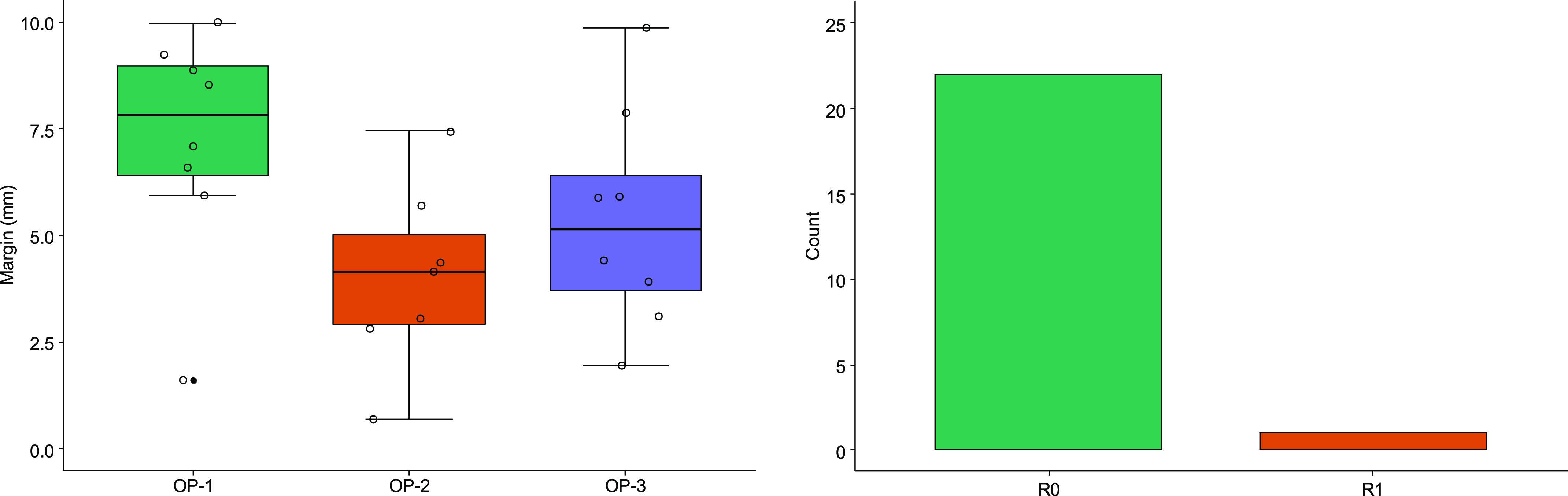
Results of the resection margin depending on the operator (left) and the number of R0 vs. R1 resections (right).

In total, 23 out of the planned 24 resections were performed, since one resection had to be cancelled by operator 2 because the tumor was not visible due to the artifacts caused by the previous resections. In 22 out of 23 resections, a negative safety margin and R0 resection was achieved by only looking at the navigation screen for guidance (Fig. 6). In one case the resection margin was below 1 mm, which was considered as an R1 resection, especially as the tumor was slightly visible on the resection specimen. Therefore, the R0 resection rate is at 95.7%. Overall, the resection margin had a median of 5.9 mm (IQR 3.5–7.7 mm).

**Fig. 7.**
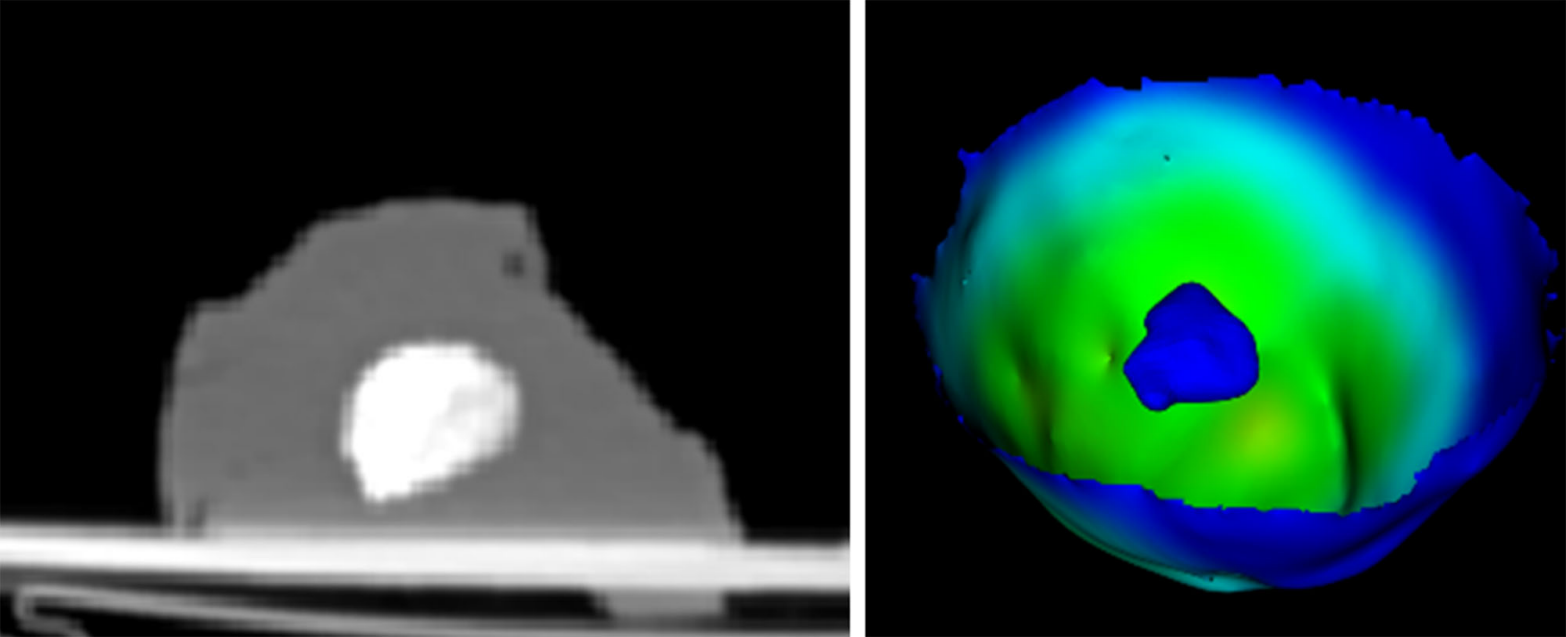
CT imaging of a resection specimen (left) and the corresponding 3D visualization of the resection margin (right).

When looking at the inter-user variability in terms of margin, surgeons 1, 2 and 3 had a median margin of 7.8 mm (IQR 6.4–9.0), 4.15 mm (IQR 2.9–5.0) and 5.1 mm (IQR 3.71–6.4) mm respectively. Even though this difference is not significant (p = 0.054), Dunn's post-hoc test shows that there is a significant difference between operator 1 and 2 (p = 0.05). When looking at the inter-user variability in terms of R0 status, the difference between the operators is not significant (p = 0.30) and only operator 2 had one R1 resection.

The median time required to plan a resection was 2 minutes (range 1–6) and 5 minutes (range 2–13) to resect the tumor. There was no significant difference between the operators neither in planning time (p = 0.8) nor in resection time (p = 0.1).

Fig. 7 shows a resection specimen under CT imaging (left) with parenchyma and the intrahepatic tumor mimic and the 3D visualization of the resection margin (right).

## IV. Discussion

In this study we evaluated a navigation method for non-anatomical liver resections based solely on navigated intra-operative US on an *ex-vivo* model. Overall, 23 tumors were resected from *ex-vivo* porcine liver with a median resection margin of 5.9 mm and an R0 resection rate of 95.7%. However, no tumor was resected with a positive margin and therefore no residual tumor remained in the parenchyma.

Compared to other studies on navigation approaches for liver surgery, we evaluated the end to end accuracy of the whole procedure rather than technical aspects like the crude registration accuracy. Consequently, this includes factors like human error and organ deformation due to US scanning and the resection itself.

We hypothesize, that deformation and tissue liquid evaporation account for the present inaccuracies. On one hand, organ deformation occurs during the resection process, because the specimen has to be pulled and moved to see the resection line for further dissection. However, the influence of this deformation is expected to be smaller compared to approaches utilizing preoperatively acquired CT/MRI imaging. Such approaches are heavily affected by the deformation occurring between the CT/MRI and the mobilization of the liver. On the other hand, electrocautery causes tissue liquids to evaporate, which leads to a shrinkage of the tissue and therefore smaller resection margins visible in the post-operative control CT.

The R0 resection rate reported in literature ranges from 66.7%–100% for these kind of resections [1]. However, this data should be interpreted carefully, as an R0 resection is not always possible due to anatomical constraints like nearby vasculature. While not directly comparable, the hypothetical R0 resection rate of 95.7% (*ex-vivo*) indicates that this tool could be useful for such resections to further increase the R0 resection rate when anatomically possible. Furthermore, the additional time required to create such a resection plan of 2 minutes (range 1–6) shows, that this approach could be applicable under clinical conditions where time is costly and rather limited.

Another application of such a navigation approach could be in cases with lesions visible only in contrast enhanced US (CEUS) only. Contrast enhancement usually lasts for maximum 1–2 minutes, which is not enough time to resect the tumor. Therefore, such a navigation approach could be used to segment the tumor on the CEUS image and then “see” it later as an overlay on the B-mode US image. This augmented US image, and the additional 3D screen would then allow the surgeon to safely remove the tumor. Similar approaches are currently carried out using image fusion with pre-operative CT/MRI onto intra-operative US, which come with technical challenges due to the registration processes and the aforementioned tissue deformations resulted since the scan was acquired [5], [26].

To further improve the navigation approach, in the first instance we aim to improve the tumor segmentation and resection visualization during the resection process. In a second instance vascular structures are to be included. Currently, the tumor is approximated by a single US slice which can be problematic in tumors with irregular shapes. This could be solved with a multi-slice segmentation method, which combines the results into a 3D mesh. Whereas the vascular structure is less important in these small resections, it is critical for larger resections, where large blood vessels must be clipped. In other instances, one might slightly reorient the resection shape to avoid such structures even if it causes a larger resection volume. In smaller resections, the blood vessels can be easily coagulated with modern resection devices.

Based on the pre-clinical data presented in this work, a clinical pilot trial with 10 patients is currently being prepared and planned to start beginning of 2020.

## V. Conclusions

Using intraoperative ultrasound-based anatomical models for navigation in liver surgery might help to achieve a higher rate of negative resection margins in non-anatomical resections of liver tumors while sparing as much healthy liver tissue as possible. This technique might be a useful tool especially for less experienced surgeons since it augments the conventional intra-operative ultrasound imaging.

## Supplementary Materials

The supplementary material consists of two videos showing parts of the planning and resection process from the ex-vivo experiments. Video 1 shows the surface scanning method with the 3D screen view and a blended view of the liver view. Video 2 shows the resection process, from the initial marking until the specimen is removed, with the view of the liver and blended view of the 3D screen.



## References

[1] D. Moris , “Anatomic versus non-anatomic resection for hepatocellular carcinoma: A systematic review and meta-analysis,” Eur. J. Surgical Oncol., vol. 44, no. 7, pp. 927–938, Jul. 2018. 10.1016/j.ejso.2018.04.018 29751946

[2] D. Moris , “Parenchymal-sparing versus anatomic liver resection for colorectal liver metastases: A systematic review,” J. Gastrointest Surgery, vol. 21, no. 6, pp. 1076–1085, Jun. 2017. 10.1007/s11605-017-3397-y 28364212

[3] D. L. Aghayan , “Laparoscopic parenchyma-sparing liver resection for colorectal metastases,” Radiol. Oncol., vol. 52, no. 1, pp. 36–41, Mar. 2018. 29520204 10.1515/raon-2017-0046 PMC5839080

[4] S. Dong, Z. Wang, L. Wu, and Z. Qu, “Effect of surgical margin in R0 hepatectomy on recurrence-free survival of patients with solitary hepatocellular carcinomas without macroscopic vascular invasion,” Medicine, vol. 95, no. 44, Nov. 2016, Art no. e5251. 10.1097/MD.0000000000005251 PMC5591133 27858885

[5] V. M. Banz , “Intraoperative image-guided navigation system: Development and applicability in 65 patients undergoing liver surgery,” Langenbeck's Archives Surgery, vol. 401, no. 4, pp. 495–502, 2016. 10.1007/s00423-016-1417-0 27122364

[6] T. Langø , “Navigated laparoscopic ultrasound in abdominal soft tissue surgery: Technological overview and perspectives,” Int. J. Comput. Assisted Radiol. Surgery, vol. 7, no. 4, pp. 585–599, Jul. 2012. 10.1007/s11548-011-0656-3 21892604

[7] T. P. Kingham, S. Jayaraman, L. W. Clements, M. A. Scherer, J. D. Stefansic, and W. R. Jarnagin, “Evolution of image-guided liver surgery: Transition from open to laparoscopic procedures,” J. Gastrointest Surgery, vol. 17, no. 7, pp. 1274–1282, Jul. 2013. 10.1007/s11605-013-2214-5 PMC3690505 23645420

[8] J. Pérez de Frutos , “Laboratory test of single Landmark registration method for ultrasound-based navigation in laparoscopy using an open-source platform,” Int. J. Comput. Assisted Radiol. Surgery, vol. 13, no. 12, pp. 1927–1936, Dec. 2018. 10.1007/s11548-018-1830-7 PMC6223760 30074134

[9] Y. Adagolodjo, N. Golse, E. Vibert, M. De Mathelin, S. Cotin, and H. Courtecuisse, “Marker-based registration for large deformations - Application to open liver surgery,” in Proc. IEEE Int. Conf. Robot. Autom., 2018, pp. 4007–4012.

[10] R. Plantefève, I. Peterlik, N. Haouchine, and S. Cotin, “Patient-specific biomechanical modeling for guidance during minimally-invasive hepatic surgery,” Ann. Biomed. Eng., vol. 44, no. 1, pp. 139–153, Jan. 2016. 26297341 10.1007/s10439-015-1419-z

[11] L. W. Clements , “Evaluation of model-based deformation correction in image-guided liver surgery via tracked intraoperative ultrasound,” J. Med. Imag., vol. 3, no. 1, Mar. 2016, Art. no. 015003. 10.1117/1.JMI.3.1.015003 PMC4804127 27081664

[12] L. W. Clements, P. Dumpuri, W. C. Chapman, B. M. Dawant, R. L. Galloway, and M. I. Miga, “Organ surface deformation measurement and analysis in open hepatic surgery: Method and preliminary results from 12 clinical cases,” IEEE Trans. Biomed. Eng., vol. 58, no. 8, pp. 2280–2289, Aug. 2011. 10.1109/TBME.2011.2146782 PMC3819167 21521662

[13] A. L. Simpson and T. P. Kingham, “Current evidence in image-guided liver surgery,” J. Gastrointest Surgery, vol. 20, no. 6, pp. 1265–1269, Jun. 2016. 10.1007/s11605-016-3101-7 PMC4970568 26956008

[14] R. L. Galloway, S. Duke Herrell, and M. I. Miga, “Image-guided abdominal surgery and therapy delivery,” J. Healthcare Eng., vol. 3, no. 2, pp. 203–228, Jun. 2012. 10.1260/2040-2295.3.2.203 PMC4112601 25077012

[15] S. Beller, M. Hünerbein, S. Eulenstein, T. Lange, and P. M. Schlag, “Feasibility of navigated resection of liver tumors using multiplanar visualization of intraoperative 3-dimensional ultrasound data,” Ann. Surgery, vol. 246, no. 2, pp. 288–294, 2007. 10.1097/01.sla.0000264233.48306.99 PMC1933548 17667508

[16] S. Beller, S. Eulenstein, T. Lange, M. Hünerbein, and P. M. Schlag, “Upgrade of an optical navigation system with a permanent electromagnetic position control: A first step towards ‘navigated control’ for liver surgery,” J. Hepatobiliary Pancreatic Surgery, vol. 16, no. 2, pp. 165–170, Mar. 2009. 10.1007/s00534-008-0040-z 19183828

[17] S. S. Chopra, M. Hünerbein, S. Eulenstein, T. Lange, P. M. Schlag, and S. Beller, “Development and validation of a three dimensional ultrasound based navigation system for tumor resection,” Eur. J. Surgical Oncol., vol. 34, no. 4, pp. 456–461, Apr. 2008. 10.1016/j.ejso.2007.07.011 17765451

[18] I. Paolucci, R. Sandu, L. Sahli, D. Candinas, S. Weber, and A. Lachenmayer, “Ex-vivo evaluation of an ultrasound-based planning and navigation method for non-anatomical liver resections,” in Proc. Tagungsband der 18. Jahrestagung der Deutschen Gesellschaft für Comput. Roboterassistierte Chirurgie, 2019, pp. 2–6.

[19] F. Lindseth, G. A. Tangen, T. Langø, and J. Bang, “Probe calibration for freehand 3-D ultrasound,” Ultrasound Med. Biol., vol. 29, no. 11, pp. 1607–1623, 2003. 14654156 10.1016/s0301-5629(03)01012-3

[20] M. Peterhans , “A navigation system for open liver surgery: Design, workflow and first clinical applications,” Int. J. Med. Robot. Comput. Assisted Surgery, vol. 7, no. 1, pp. 7–16, 2011. 10.1002/rcs.360 21341357

[21] L. Sahli, S. Weber, and I. Paolucci, “Liver surface reconstruction from navigated ultrasound during image-guided liver surgery acquisition surface contact detection reconstruction,” in Proc. Tagungsband der 17. Jahrestagung der Deutschen Gesellschaft für Comput. Roboterassistierte Chirurgie, 2018, pp. 1–4.

[22] M. M. Breunig, H.-P. Kriegel, R. T. Ng, and J. Sander, “LOF,” in Proc. SIGMOD Int. Conf. Manage. Data, 2000, vol. 29, no. 2, pp. 93–104.

[23] H. Hoppe, Surface Reconstruction From Unorganized Points. Seattle, WA, USA: Univ. of Washington Press, 1995.

[24] C. Rother, V. Kolmogorov, and A. Blake, “‘GrabCut’: interactive foreground extraction using iterated graph cuts,” in Proc. ACM SIGGRAPH, Aug. 2004, vol. 23, no. 3, pp. 309–314.

[25] A. Dinno, “Nonparametric pairwise multiple comparisons in independent groups using Dunn's test,” Stata J. Promoting Commun. Statist. Stata, vol. 15, no. 1, pp. 292–300, Apr. 2015.

[26] L. M. Pak , “Utility of image guidance in the localization of disappearing colorectal liver metastases.,” J. Gastrointest Surgery, vol. 23, no. 4, pp. 760–767, Apr. 2019. 10.1007/s11605-019-04106-2 PMC6717434 30680630

